# Impact of meteorological factors and population density on COVID-19 pandemic in Saudi Arabia

**DOI:** 10.1016/j.sjbs.2022.103545

**Published:** 2022-12-23

**Authors:** Khalid J. Alzahrani, Nadim Sharif, Afsana Khan, Hamsa Jameel Banjer, Anowar Khasru Parvez, Shuvra Kanti Dey

**Affiliations:** a Department of Clinical Laboratories Sciences, College of Applied Medical Sciences, Taif University, P.O. Box 11099, Taif 21944, Saudi Arabia; b Department of Microbiology, Jahangirnagar University, Savar, Dhaka 1342, Bangladesh; c Department of Statistics, Jahangirnagar University, Savar, Dhaka 1342, Bangladesh

**Keywords:** COVID-19, Weather, Population, Variant of concern, Vaccination, Saudi Arabia

## Abstract

Transmission and increase in cases and fatalities of coronavirus disease-2019 (COVID-19) are significantly influenced by the parameters of weather, human activities and population factors. However, study gap on the seasonality of COVID-19 and impact of environmental factors on the pandemic in Saudi Arabia is present. The main aim of the study is to evaluate the impact of environment on the COVID-19 pandemic. Data were analyzed from January 2020 to July 2021. The generalized estimating equation (GEE) was used to determine the effect of environmental variables on longitudinal outcomes. Spearman's rank correlation coefficient (*r_s_*) was used to analyze the impact of different parameters on the outcome of the pandemic. Multiple sequence alignment was performed by using ClustalW. Vaccination and fatalities (*r*_s_ = −0.85) had the highest association followed by vaccination with cases (*r*_s_ = −0.81) and population density with the fatalities (*r_s_* = 0.71). The growth rate had the highest correlation with sun hours (*r*_s_ = −0.63). Isolates from variant of concern alpha and beta were detected. Most of the reference sequences in Saudi Arabia were closely related with B.1.427/429 variant. Clade GH (54%) was the most prevalent followed by O (27%), GR (9%), G (6%), and S (4%), respectively. Male to female patient ratio was 1.4:1. About 95% fatality and hospitalization were reported in patients aged >60 years. This study will create a comprehensive insight of the interaction of environmental factors and the pandemic and add knowledge on seasonality of COVID-19 in Saudi Arabia.

## 1 Introduction

SARS-CoV-2 from family *Coronaviridae* has started the COVID-19 pandemic (Chen et al., 2020, Peeri et al., 2020, Zhang et al., 2020). The first human coronavirus (strain 229E and OC43) were isolated during 1953 from patients with common cold symptoms (Matoba et al., 2015). Local and limited outbreaks by other members of *Coronavirus* such as SARS-CoV, HKU1, and MERS-CoV have been reported during 2003–2005 and 2012 (Chen et al., 2020, Matoba et al., 2015, Peeri et al., 2020, Zhang et al., 2020). As of January 23, 2022, SARS-CoV-2 has infected about 350 million and killed 56 million people worldwide (GitHub COVID-19 database, 2022, Worldometer, 2022, World Health Organization, 2022).

Patients with COVID-19 have reported different types of clinical symptoms (Huang et al., 2020, Matoba et al., 2015, Zhang et al., 2020). According on the period of illness and health outcome, clinical symptoms might be classified as severe, mild, or asymptomatic (Alsofayan et al., 2020, Huang et al., 2020, Kannan et al., 2020, Tian et al., 2020, Yuki et al., 2020). Distribution of frequency of asymptomatic and symptomatic patients largely depends on the circulating variants (Huang et al., 2020, Kannan et al., 2020, Tian et al., 2020). Nearly 80–85% of the symptomatic patients develop mild diseases (Alsofayan et al., 2020, Kannan et al., 2020, Tian et al., 2020, Yuki et al., 2020). In mild cases clinical symptoms like fever, chills, cough, and sore throat are common, followed by headache, tremors, loss of smell and taste, and sometimes muscle pain. Asymptomatic people or patients with moderate symptoms have high recovery rate. Symptoms like breathing problem, pneumonia, renal failure, and multiple organ failure have been recorded among patients with severe conditions (Alsofayan et al., 2020, Huang et al., 2020, Kannan et al., 2020, Tian et al., 2020, Yuki et al., 2020).

The first case of COVID-19 in Saudi Arabia was detected on early March 2020 (Worldometer, 2022, World Health Organization, 2022). Till July 29, 2021, about 500,000 cases and 8,200 fatalities of COVID-19 have been reported from Saudi Arabia (Worldometer, 2022, World Health Organization, 2022). The first wave of COVID-19 in Saudi Arabia was apparently confined during the period of April to September 2020. About 70% of the cases and fatalities were detected in the first wave (Worldometer, 2022, World Health Organization, 2022). Among the 13 regions, the highest number of cases has been reported from Makkah (∼125,000 cases, 24%) followed by Riyadh (∼123,000 cases, 22%), Eastern Province (112,000 cases, 22%), Asir (37,556 cases, 7.1%), Madinah (37,357 cases, 7.1%), Qasim (19,106 cases, 3.7%), Jizan (18,140 cases, 3.5%) and Ha'il (10,330 cases, 2%), respectively. The highest number of fatality was reported from Riyadh (∼1,500) followed by Eastern Province (1,218) and Asir (596), respectively. Total population of Saudi Arabia is 35,393,638 with a population density of 16 person/Km^2^. As of July 29, 2021, a total of ∼4,200,000 (12.5%) people have been vaccinated with two doses (Algahtany et al., 2018, Worldometer, 2022, World Health Organization, 2022).

SARS-CoV-2 is a positive sense single stranded RNA (ssRNA) virus with a linear, non-segmented, genome of ∼30,000 bases (Chan et al., 2020, Sharif and Dey, 2020). The proteome of SARS-CoV-2 is comprised of 4 structural proteins and about 16 non-structural proteins (Chan et al., 2020, Sharif and Dey, 2020, Khailany et al., 2020, Davidson et al., 2020). Alteration in the spike protein’s receptor binding sites (RBD) due to mutations is one of the major factors of emergence of new variants (Chan et al., 2020, Davidson et al., 2020, Khailany et al., 2020, Lu et al., 2020, Sharif and Dey, 2020). Mutational events at the virus genome are influenced by host and environmental parameters. The immune system, coinfection, vaccine and antivirals pressure influence coronavirus genome changes inside the host body (Sharif and Dey, 2021). In addition, different factors form environment such as ultra violet rays, ambient temperature, and humidity may have significant roles in the evolution of new variants (Sharif and Dey, 2021).

Weather may have profound impact on the dissemination of COVID-19 (Bashir et al., 2020, Sharif and Dey, 2021). Relative humidity, UV index, wind speed, ambient air temperature, rain fall, and precipitation are the major environmental factors that may have shaped the COVID-19 pandemic (Bashir et al., 2020, Bilal et al., 2021, Sharif and Dey, 2021, Sharif et al., 2021a, Sharif et al., 2021b, White et al., 2020). Among other factors, vaccination, the likelihood to follow preventive measures, health policies, population density, mobility and transportation of patients, and gatherings during national and international events have contributed significantly in the transmission and continuation of the COVID-19 pandemic worldwide (Bashir et al., 2020, Bilal et al., 2021, Sharif and Dey, 2021, Sharif et al., 2021a, Sharif et al., 2021b, Tan et al., 2005, White et al., 2020, Xie and Zhu, 2020).

The main objective of this study is to evaluate the association of the population density, meteorological parameters and sociodemographic factors with the COVID-19 pandemic in Saudi Arabia. Other goals are to investigate the relationship between mutations in SARS-CoV-2 genome and regulatory factors such as weather parameters and host factors. This study also analyzed the impact of human activities on the COVID-19 pandemic in Saudi Arabia. This study will create a cumulative insight of circulating variants of SARS-CoV-2 in Saudi Arabia.

## 2 Materials and methods

### 2.1 Study areas and time frame

This study was conducted to evaluate the effect of regulatory factors including parameters of weather and human activities on the COVID-19 pandemic in Saudi Arabia. This study included data and parameters during January 2020 to July 2021. Data were collected from 13 regions of Saudi Arabia including Asir (19°N to 43°E), Al Bahah (20°N to 41°E), Al Jawf (29°30′N to 39°30′E), Eastern Province (23°N to 49°E), Ha'il (27°7′N to 41°9′E), Jizan (17°1′N to 42°7′E), Makkah (21°5′N to 41°9′E), Madinah (24°8′N to 39°3′E), Najran (18°3′N to 45°6′E), Northern Borders (30°N to 42°8′E), Qasim (26°2′N to 43°5′E), Riyadh (22°7′N to 46°2′E), and Tabuk (28°2′N to 37°6′E).

### 2.2 Study data collection and data availability

An unbiased approach was used to collect the data on COVID-19 in Saudi Arabia from the official website of the government (https://covid19.moh.gov.sa/). Further, data on cases, fatalities, number of tests, total population, and population density were collected, evaluated and analyzed from different sources including WHO dashboard (https://covid19.who.int/region/emro/country/sa), Worldometers (https://www.worldometers.info/coronavirus/country/saudi-arabia/), COVID-19 Tracker (https://graphics.reuters.com/world-coronavirus-tracker-and-maps/countries-and-territories/saudi-arabia/) (Worldometer, 2022, World Health Organization, 2022).

Data on meteorological parameters such as temperature (°C), humidity, UV intensity, rainfall (mm), wind velocity (km/h), were collected, analyzed and included from Department of Meteorology, Saudi Arabia (https://weather.kau.edu.sa/Default-155001-EN), and Weather forecast Saudi Arabia (https://www.weather-atlas.com/en/saudi-arabia).

Whole genomes of SARS-CoV-2 in Saudi Arabia were retrieved from GISAID (https://www.gisaid.org/) (GISAID, 2022). Human activities including data on national and international movement, religious and social gatherings, duration of lockdown and sport events were included in this study. Further, data on vaccination was collected and analyzed from Our World in Data (https://ourworldindata.org/covid-vaccinations?country=OWID_WRL∼SAU).

### 2.3 Whole genome, mutational and phylogenomic analysis of SARS-CoV-2

Full length genome sequence of SARS-CoV-2 isolated in Saudi Arabia were analyzed by using MEGA X (Dey et al., 2020, Kumar et al., 2018, Sharif et al., 2020). Homology analysis was conducted by using the BLASTn program (https://blast.ncbi.nlm.nih.gov/Blast.cgi). Multiple sequence alignment (MSA) of the isolates were conducted by using ClustalW Multiple Alignment algorithm by using the BioEdit 7.2.6 software (Dey et al., 2020, Sharif et al., 2020). NC_045512/Wuhan-Hu-1 was used as the reference strains. Clades definition was determined by the presence of specific genomic markers (Sharif and Dey, 2021, Sharif et al., 2021b). Phylogenomic tree was built by using Kaimura-2-parameter algorithm by the maximum composite likelihood method. Bootstrap value of 1000 was used to evaluate the reliability of the tree. Further, using the reference genome (NC_045512/Wuhan-Hu-1), deletion, indels and substitution point mutations in the study sequences were analyzed by the MEGA 10 software.

### 2.4 Statistical analyses

Appropriate and unbiased statistical methods were implemented to evaluate and analyze the collected data. The generalized estimating equation (GEE) was used to determine the effect of environmental variables on longitudinal outcomes, the monthly incidence rate and monthly case-fatality rate. The GEE approach was used for analyzing the longitudinal outcomes, and didn’t require distributional assumption on the outcomes. We focused on the environmental variables including temperature, snowfall, sun hours, humidity, rainfall, and wind speed. Further, Spearman's rank correlation coefficient (*r*_s_) was performed to evaluate the impact of different parameters on the outcome of COVID-19 in Saudi Arabia (Sharif and Dey, 2021, Sharif et al., 2021b). Moreover, regression analysis was used to determine the correlation between host factors and mutation frequency of the virus. Spearman's rank correlation coefficient (*r*_s_) was determined by using the following equation:

$$r_{s}=1-6\frac{\sum d_{i}^{2}}{n(n^{2}-1)}$$

Here ‘n’ = the number of observations, ‘d_i_’ = the difference between the ranks and *r_s_* = Spearman’s correlation coefficient.

## 3 Results

### 3.1 Spatial and temporal trends of COVID-19 in Saudi Arabia

**Fig. 1:**
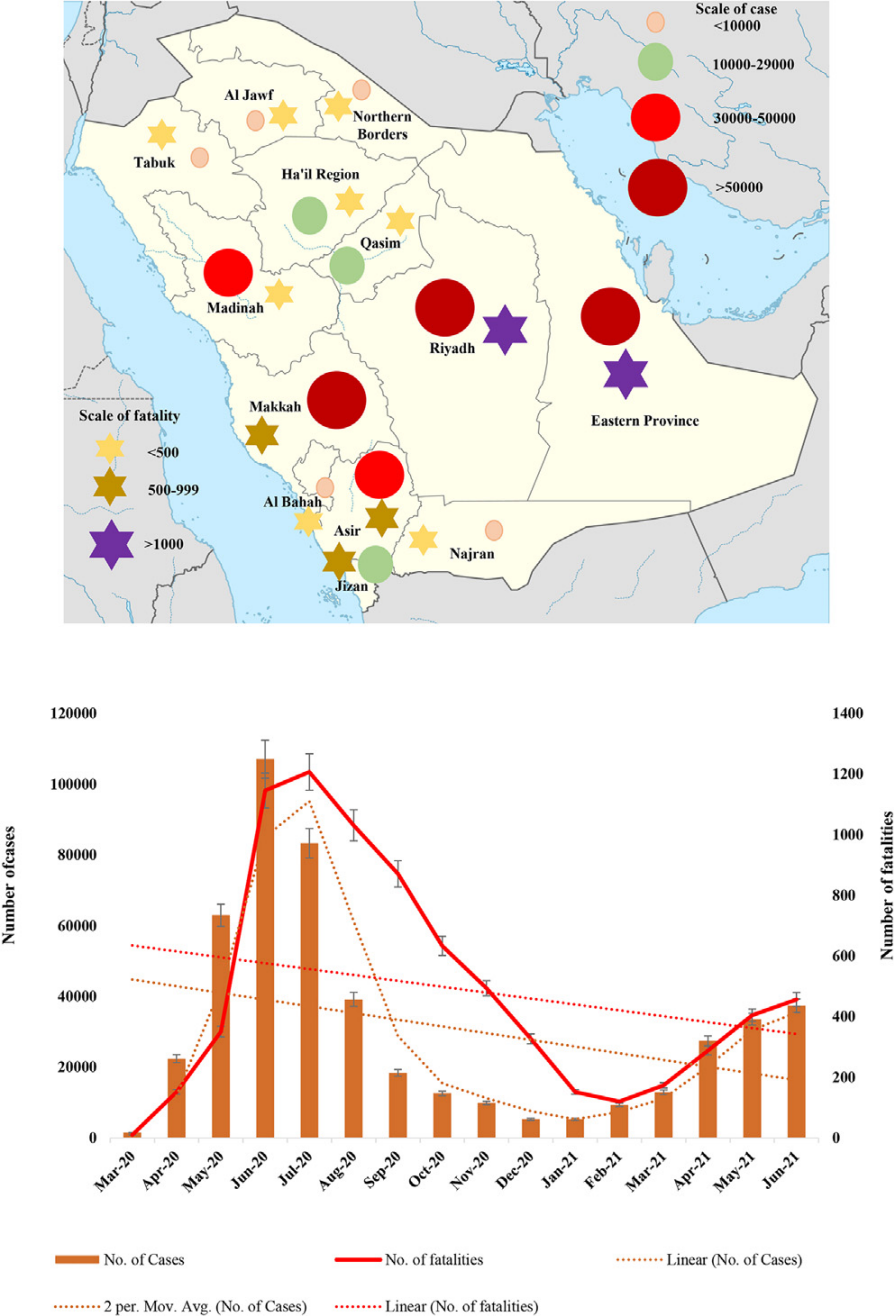
(A) Spatial distribution of COVID-19 cases and fatalities in 13 regions in Saudi Arabia during January 2020 to July 2021; (B) Monthly distribution of cumulative cases and fatalities of COVID-19 in Saudi Arabia.

The highest number of cases and fatalities in Saudi Arabia was recorded during the first wave from April 2020 to September 2020. However, from April 2021, both the number of cases and fatalities began to rise again (Fig. 1A–B). About 136 governorates are distributed into 13 provinces in Saudi Arabia. Among the major cities and towns the highest number of COVID-19 case was recorded from Riyadh (∼100,000) followed by Jeddah (∼100,000), Makkah (∼100,000) and Madinah (∼100,000), respectively. On the contrary the lowest number of case was recorded in Harad (1), followed by As Sulaymaniyyah (1), Samudah (3), and Abu 'urwah (6), respectively. Case fatality rate has remained highest in Al Jawf (512 per 10,000 cases) followed by Makkah (391 per 10,000 cases), Northern Borders (242 per 10,000 cases) and Ha'il (197 per 10,000 cases), respectively (Table 1). However, the death rate was the highest in Jizan (562 death per 100,000 persons).

**Table 1:** Cumulative COVID-19 cases and fatalities, case fatality rate, total population and vaccination frequency in 13 different provinces in Saudi Arabia.

Regions	No. of cases	No. of fatalities	Case fatality rate per ten thousand cases	Death rate per 100,000 persons	Total population	Vaccinated population %
Asir	37,556	596	21	27	2,211,875	
Al Bahah	6,514	86	132	82	104,266	
Al Jawf	2,301	118	512	23	508,475	
Eastern Province	112,317	1,218	108	25	4,900,325	
Ha'il	10,330	203	197	34	605,930	
Jizan	18,140	591	173	562	105,193	12.3%
Makkah	123,248	326	391	16	2,042,000	(4,200,000)
Madinah	37,357	55	15	5	1,183,205	
Najran	9,209	104	113	21	505,652	
Northern Borders	4,546	110	242	29	375,310	
Qasim	19,106	270	141	22	1,215,858	
Riyadh	122,509	1,495	122	19	7,676,654	
Tabuk	7,736	100	129	11	910,030	

### 3.2 Analyses of population factor and parameters of weather

Various meteorological parameters including ambient temperature, UV index, percentage of relative humidity, velocity of wind, amount of rainfall, and sun hours were included in this study. Three values of ambient temperature were recorded from January 2020 to July 2021 in 13 provinces in Saudi Arabia. The mean value of maximum temperature was 39 °C, and varied between 28 °C and 54 °C in thirteen provinces in Saudi Arabia. The mean value of temperature average was 29 °C and temperature minimum was 18 °C in Saudi Arabia the COVID-19 pandemic (Supplementary Fig. 1A–M). The average value of three temperatures fluctuated ±8 °C in 13 provinces. A peak of ambient temperatures were apparently confined within a period of five months (May–September) during 2020–2021 (Supplementary Fig. 1A–M). The period of the first peak of the COVID-19 pandemic overlapped the peak of temperature in 2020, and another peak of the cases is on the rise from April 2021 (Fig. 1A–B).

Both the sun hours and UV index were documented from 13 provinces in Saudi Arabia. The average UV index in Saudi Arabia ranged from moderate to extreme (3 to >11) during the study period (Supplementary Fig. 2A). The highest UV index average was recorded 16 in Eastern province, Ha’il and Al Jawf AND the lowest UV index average was reported 4 in Makkah during October 2020 (Supplementary Fig. 2A). Distinct peak of UV index couldn’t be defined during this study.

Average value of relative humidity in Saudi Arabia varied from 10% to 75% during January 2020 to July 2021. A peak of relative humidity was detected during October 2020 to February 2021. The highest average relative humidity was recorded in Eastern province (65%) and Northern Borders (55%). The lowest average relative humidity was recorded in Madinah (35%) and Ha’il (40%) (Supplementary Fig. 2B). Further, the average barometric pressure was about 1 atm during January 2020 to July 2021 in Saudi Arabia.

**Fig. 2:**
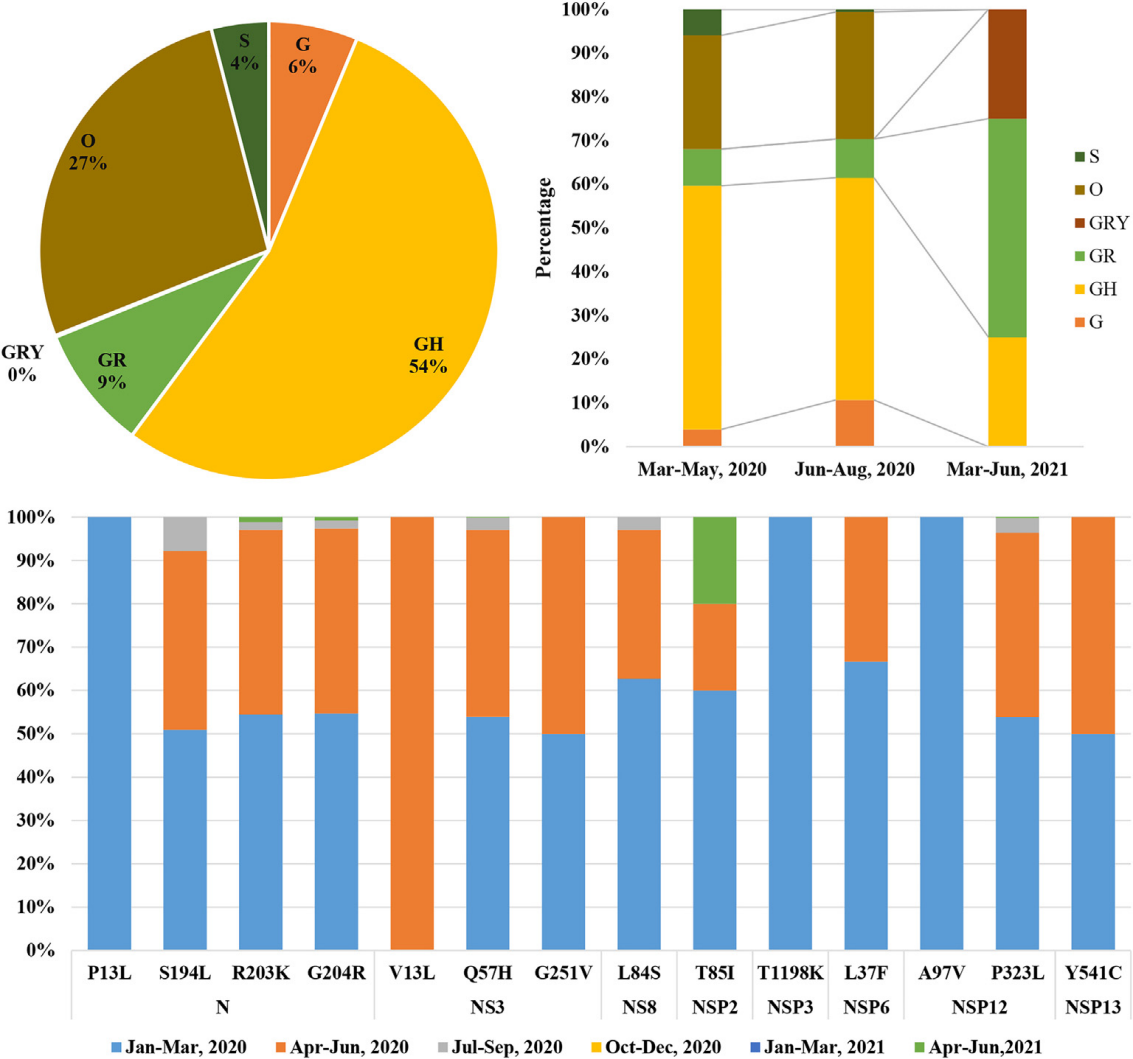
Upper left panel is showing the overall frequency distribution of clades of SARS-CoV-2 in Saudi Arabia, Upper right panel is showing the temporal distribution of clades of SARS-CoV-2 in Saudi Arabia, Lower panel is representing the temporal frequency distribution of point mutations among isolates of SARS-CoV-2 in Saudi Arabia.

**Fig. 3:**
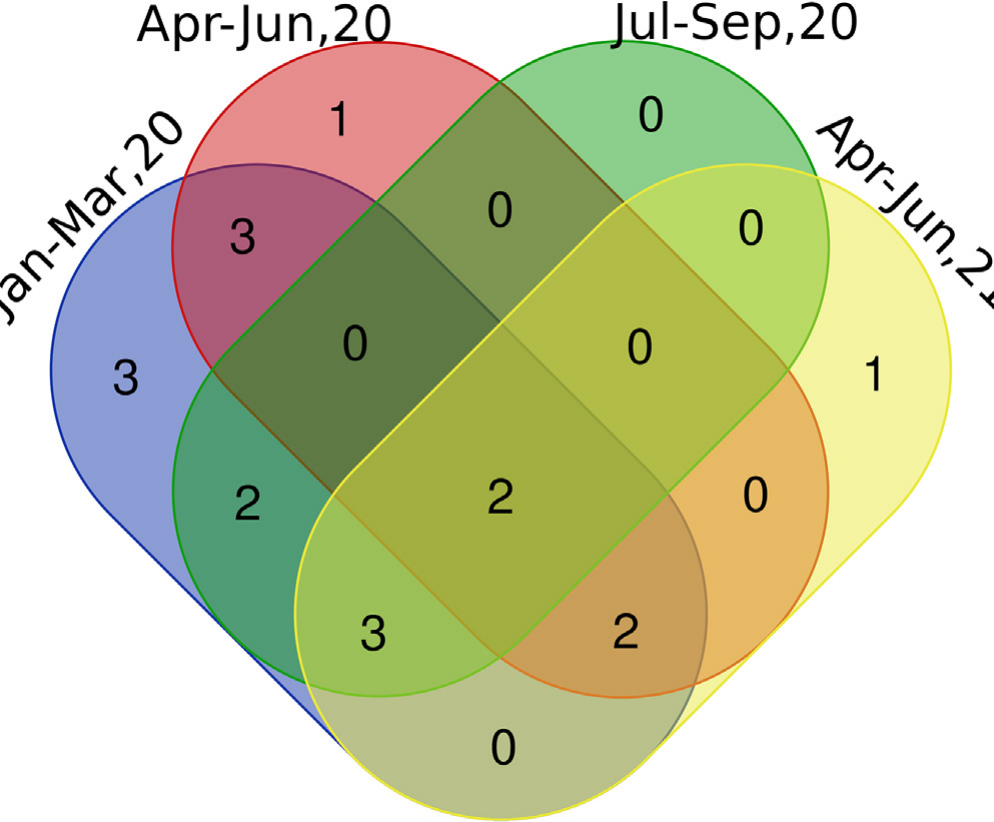
Venn diagram representing the common substitution point mutations of study isolates in Saudi Arabia during March 2020 to July 2021.

Average wind velocity in Saudi Arabia during the pandemic was analyzed. The average wind velocity varied from 5 km/h to 33 km/h. The highest wind speed average was reported in Eastern province (19 km/h) and Ha’il (15 km/h), while the lowest wind speed average was reported in Jizan (6 km/h), and Qasim (9 km/h) (Supplementary Fig. 2C).

The data on average rainfall and snowfall in Saudi Arabia were included in this study. However, the data on both of them were not persistent for all 13 provinces.

Population factors including population density and total population in Saudi Arabia were also included in this study. Riyadh (7,676,654) had the highest number of residents followed by Eastern province (4,900,325), Asir (2,211,875), and Makkah (2,042,000), respectively. Riyadh (11,000 residents/mile^2^) had the most number of residents living per square mile followed by Qasim (6,700 residents/mi^2^), Makkah (5,700 residents/mi^2^), Jizan (350 residents/mi^2^), Al Bahah (120 residents/mi^2^), Najran (87 residents/mi^2^), Madinah (36 residents/mi^2^), and Eastern province (19 residents/mi^2^), respectively. Riyadh is the capital and the place of most of the national and international transportations in Saudi Arabia.

### 3.3 Determination of correlation between weather and COVID-19 in Saudi Arabia

Overall, the incidence of COVID-19 increased over time in Saudi Arabia. Adjusted for the other variables, for every 1 °C increase in temperature, the incidence decreased by 2 per 10,000 population on average; for every 1 unit of increase of UV index, the incidence decreased by 3.8 per 10,000 population on average; for every 10% decrease of relative humidity, the incidence increased by 4.1 per 10,000 population on average. Three time frames including the first day of the report of cases, after 7 days and after 14 days of the report of cases were used. Among the cases and parameters of weather, relative humidity (RH) on the day of the report of case had the highest correlation with number of case (*r*_s_ = −0.62), followed by minimum temperature (min temp) on the day (*r*_s_ = −0.61), average temperature after 7 days (*r*_s_ = −0.53), respectively (Table 2). Further, the growth rate had the highest correlation with sun hours on the day of the report of the case (*r*_s_ = −0.63) followed by sun hours after 7 days (*r*_s_ = −0.51) and minimum temperature on the day (*r*_s_ = −0.42) (Table 2). The fatalities of COVID-19 had the highest correlation with RH on the day (*r*_s_ = −0.55). Case fatality rate had the strongest correlation with RH (*r_s_* = −0.62) and maximum temperature (*r*_s_ = −0.61) on the day (Table 2). Wind velocity was positively related with the outcome of the COVID-19 pandemic in Saudi Arabia, which predicted that with the increase of wind velocity the cases increased. Both the total population and population density of different cities of Saudi Arabia were positively related with the outcome of COVID-19 pandemic. Total population and population density had the strongest association with the fatalities (*r_s_* = 0.67 and *r_s_* = 0.71, respectively). The frequency of vaccination in Saudi Arabia had the strongest association with fatalities (*r*_s_ = −0.85) and cases (*r*_s_ = −0.81) (Table 2).

**Table 2:** Spearman’s correlation among meteorological parameters and COVID-19 pandemic in Saudi Arabia.

Environmental factors	COVID-19 cases	Growth rate	COVID-19 fatalities	Case fatality rate
Max temp on the day	−0.24	−0.012	−0.31*	−0.61*
Max temp after 7 days	−0.002	−0.05	−0.19*	−0.03
Max temp after 14 days	−0.014	−0.03	−0.14	−0.001
Avg temp on the day	−0.41**	−0.12	−0.27**	−0.14
Avg temp after 7 days	−0.53*	−0.24	−0.39	−0.09*
Avg temp after 14 days	−0.31	−0.18**	−0.12	−0.31
Min temp on the day	−0.61*	−0.42*	−0.36*	−0.40
Min temp after 7 days	−0.14	−0.02	−0.38*	−0.009
Min temp after 14 days	−0.19	−0.10	−0.09	−0.13*
UV index on the day	−0.18*	−0.31*	−0.12*	−0.40*
UV index after 7 days	−0.24**	−0.14	−0.09	−0.23*
UV index after 14 days	−0.27*	−0.03*	−0.16**	−0.35
Sun hours on the day	−0.39*	−0.63*	−0.29	−0.26*
Sun hours after 7 days	−0.25	−0.51*	−0.21**	−0.16*
Sun hours after 14 days	−0.32	−0.28	−0.19	−0.13*
RH on the day	−0.62**	−0.17	−0.55*	−0.62*
RH after 7 days	−0.33	−0.16	−0.34	−0.27*
RH after 14 days	−0.29*	−0.41	−0.11**	−0.07
Rainfall on the day	−0.14*	−0.09*	−0.25	−0.12*
Rainfall after 7 days	0.04	0.01	−0.03	0.11
Rainfall after 14 days	−0.001	−0.004	−0.002	−0.17*
Wind speed on the day	0.21*	0.16*	0.26**	0.32*
Wind speed after 7 days	0.46*	0.34*	0.17*	0.37*
Wind speed after 14 days	0.11	0.06	0.001	0.17
Total population	0.61**	0.54**	0.67*	0.38**
Population density	0.57*	0.41*	0.71*	0.14*
Vaccination	−0.81**	−0.54*	−0.85*	−0.76*

**, * stands for 1%, 5% level of significance.

### 3.4 Analyses of temporal distribution of clades and variants of SARS-CoV-2 in Saudi Arabia

About 950 whole genome of SARS-CoV-2 were retrieved and analyzed from Saudi Arabia. Frequency distribution of the variants and clades were analyzed. Among the isolates, clade GH (54%) was the most prevalent followed by O (27%), GR (9%), G (6%), and S (4%), respectively (Fig. 2). The prevalence of clade GH remained higher during March to August 2020 and the frequency of clade GR had increased during March to July 2021. Among the variants of concern (VOC), one isolate of alpha variant and one isolate of beta variant were detected in April 2021. Among the study whole genome in Saudi Arabia, other VOC including gamma or delta was absent.

### 3.5 Phylogenomic and mutational analyses of SARS-CoV-2 in Saudi Arabia

About 950 isolates of SARS-CoV-2 were analyzed for substitution point mutation, insertion and deletion in Saudi Arabia. Deletion of bases at the 5′ untranslated regions and 3′ untranslated regions were prevalent in most of the isolates. However, at spike protein, the frequency of any type of mutation was low. Among the substitution point mutations at spike protein, D614G was the most prevalent (85%). Other point mutations including H69del, N501T, and V1228L were found in rare. However, substitution at receptor binding domain and other mutations including K417N, N439K, S477N, T478I, E484K, E484Q, and N501Y were absent in the isolates in Saudi Arabia.

Substitution point mutation and deletions at nucleocapsid (N) and non-structural proteins were also detected. At N region, P13L, S194L, R203K and G204R were found (Fig. 2). Among the non-structural proteins, substitutions were detected at NS3, NS8, NSP2, NSP3, NSP6, NSP12 and NSP13 regions. Among 23 substitution point mutations of all the isolates in Saudi Arabia, D614G at spike and P323L at NSP12 were detected consistently during the pandemic (Fig. 3).

**Fig. 4:**
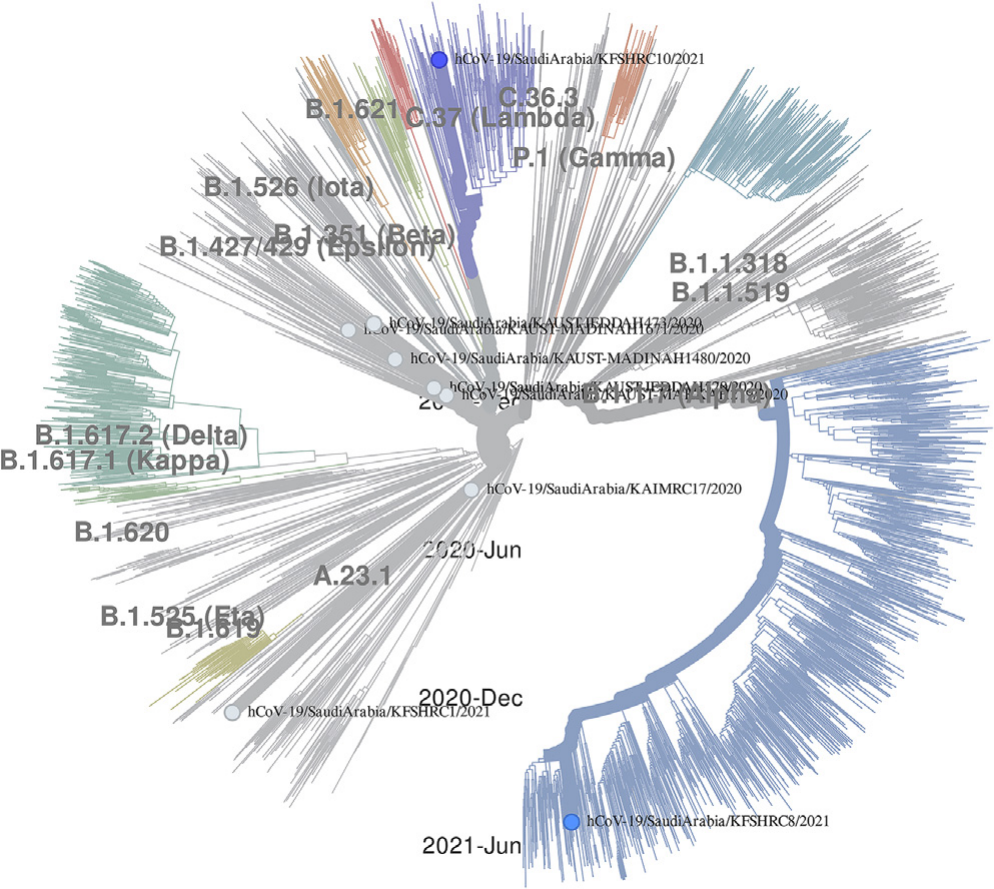
Phylogenomic tree of isolates of SARS-CoV-2 from Saudi Arabia and reference sequences around the world. The tree was built by using maximum likelihood model by using Kaimura-2-parameter algorithm. Bootstrap value of 1000 was used to test the reliability of the branches.

Reference sequences from Saudi Arabia were compared with all the representative reference sequences in the world. Most of the reference sequences from Saudi Arabia were closely related with B.1.427/429 variant. One isolate from early 2020 and another one from late 2020 were closely related with A.23.1, while another isolate was closely related with C.37 variant (Fig. 4).

### 3.6 Distribution of cases and fatalities among the patients

**Fig. 5:**
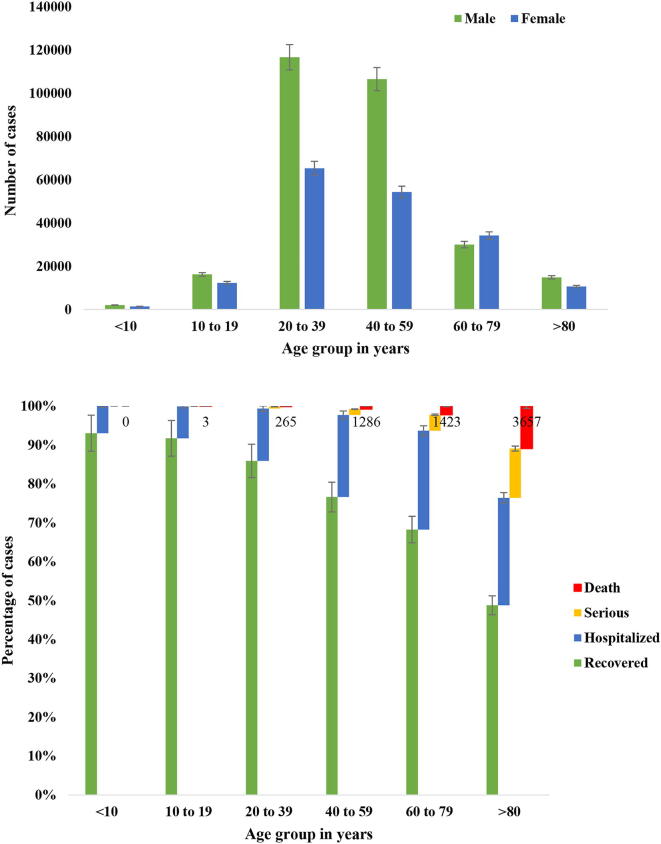
Upper panel represented the gender distribution of cases of COVID-19 and lower panel represented the age distribution of health outcome among patients of COVID-19 in Saudi Arabia.

We included data on the distribution of age and sex of the patients of COVID-19 in Saudi Arabia. Male was the prevalent sex group among the infected patients (Fig. 5). The ratio of male to female patients was 1.4:1. However, the number of female patient was prevalent in age group 60–79. The percentage of recovery declined with increasing age of the patients. The frequency of hospitalization, severity of disease and deaths were most prevalent in patients aged above 60 years in Saudi Arabia (Fig. 5).

### 3.7 Impact of host factors on COVID-19 pandemic

Univariate regression analysis was conducted to evaluate the impact of hosts on mutations of SARS-CoV-2 in Saudi Arabia. The risk of substitution and deletion at S [OR: 1.8 (95% CI 0.85–2.6), *p-value* 0.001] and N [OR: 2.4 (95% CI 1.7–3.8), *p-value* 0.01] protein increased with population density. Vaccination reduced the risk of mutations at most of the sites of genome SARS-CoV-2 (Table 3). Increase of case number contributed to the risks of mutation significantly at S [OR: 2.3 (95% CI 1.6–3.8), *p-value* 0.001], E [OR: 3.1 (95% CI 1.8–4.3), *p-value* 0.05], M [OR: 2.2 (95% CI 1.1–3.9), *p-value* 0.04], N [OR: 1.9 (95% CI (0.7–3.3), *p-value* 0.01], and nonstructural proteins NS3 [OR: 2.5 (95% CI 1.4–4.1), *p-value* 0.05], NSP2 [OR: 2.4 (95% CI (0.5–4.2), *p-value* 0.005], and NSP12 [OR: 2.4 (95% CI 0.8–4.8), *p-value* 0.03]. Further, age of the residents increased the risks of mutation at S [OR: 1.6 (95% CI 0.6–2.7), *p-value* 0.001] and NSP3 [OR: 2.6 (95% CI 1.7–4.9), *p-value* 0.001]. Gender was also involved with higher risk of mutations at S [OR: 1.2 (95% CI 0.2–3.8), *p-value* 0.001] and E [OR: 1.8 (95% CI 0.7–4.1), *p-value* 0.05] proteins (Table 3).

**Table 3:** Regression analysis of host factors and frequency of substitution point mutations and deletion at structural and nonstructural proteins of SARS-CoV-2 in Saudi Arabia.

Mutation site	OR (95% CI),							*p-value*
	Population density	Total population	Vaccination conditions	Case number	Fatality rate	Age	Gender	
S	1.8 (0.85–2.6)	2.7 (1.3–3.9)	0.49 (0.2–1.5)	2.3 (1.6–3.8)	0.9 (0.25–2.9)	1.6 (0.6–2.7)	1.2 (0.2–3.8)	0.001
E	1.2 (0.6–2.7)	1.7 (0.5–2.9)	1.4 (0.8–2.1)	3.1 (1.8–4.3)	0.7 (0.1–2.2)	1.4 (0.4–3.8)	1.8 (0.7–4.1)	0.05
M	1.6 (0.8–3.5)	1.1 (0.6–2.2)	0.5 (0.2–1.2)	2.2 (1.1–3.9)	1.3 (0.6–3.8)	1.1 (0.5–2.9)	1.4 (0.5–4)	0.04
N	2.4 (1.7–3.8)	1.5 (0.7–2.3)	0.2 (0.1–0.9)	1.9 (0.7–3.3)	1.6 (0.9–4.1)	1.7 (0.5–2.9)	1.5 (0.9–4.2)	0.01
NS3	0.9 (0.6–2.6)	1.2 (0.4–2.2)	0.5 (0.1–1.6)	2.5 (1.4–4.1)	0.5 (0.1–2.3)	1.9 (1.1–4.8)	1.9 (1–4.5)	0.05
NS8	0.7 (0.5–1.9)	0.9 (0.4–1.8)	0.1 (0.04–1.2)	1.9 (0.6–3.8)	1.7 (0.7–3.5)	1.7 (0.8–4.1)	1.7 (0.8–3.8)	0.07
NSP2	0.3 (0.1–1.3)	1.1 (0.6–2.5)	0.7 (0.2–2.1)	2.4 (0.5–4.2)	1.2 (0.4–3.4)	0.3 (0.1–3.1)	0.6 (0.1–2.2)	0.005
NSP3	1.2 (0.6–2.6)	1.4 (0.6–2.7)	0.2 (0.02–1.4)	1.7 (0.7–3.8)	0.7 (0.2–2.8)	2.6 (1.7–4.9)	1.6 (0.5–3.8)	0.001
NSP6	0.8 (0.2–2.2)	1.2 (0.9–2.1)	0.3 (0.07–0.9)	1.5 (0.6–3.1)	0.6 (0.1–3.9)	1.4 (0.8–4.7)	1.5 (0.7–3.8)	0.004
NSP12	0.9 (0.5–2.1)	0.7 (0.4–2.1)	0.9 (0.4–1.7)	2.4 (0.8–4.8)	1.3 (0.4–3.2)	1.1 (0.5–4.2)	0.8 (0.2–3.7)	0.03
NSP13	1.2 (0.6–1.8)	1.4 (0.5–2.2)	0.6 (0.1–1.8)	0.9 (0.1–3.8)	0.8 (0.2–3.1)	1.3 (0.5–3.9)	0.7 (0.1–4.2)	0.001

## 4 Discussion

Emergence and circulation of new variants of COVID-19 with increased transmission rate have initiated new waves by infecting and killing millions of people globally (Roy et al., 2021). Severity and outcome of a pandemic like COVID-19 are regulated by various factors such as pathogen characteristics, hosts parameters, environmental conditions, intervention and preventive measures (Sharif and Dey, 2021, Sharif et al., 2021a, Sharif et al., 2021b, Şahin, 2020). Meteorological parameters influence the transmission of COVID-19. Host and population factors have significant influences on demining the severity of cases and outcome of the pandemic (Şahin, 2020, Sharif and Dey, 2021, Sharif et al., 2021a, Sharif et al., 2021b). Several studies on the impact of regulatory factors including weather, population and hosts have been conducted in China, the USA, Indonesia, Bangladesh, Japan and Turkey (Bashir et al., 2020, Şahin, 2020, Sajadi et al., 2020, Sharif et al., 2021a, Sharif et al., 2021b, Sharif et al., 2021d, Sharif and Dey, 2021, Tosepu et al., 2020). However, significant research gap on the association of meteorological parameters, and population factors with the COVID-19 pandemic is present in hot temperate regions like Saudi Arabia. Therefore, we conducted this study to understand the effects of weather on COVID-19 and seasonality of the pandemic. Significant correlation between factors of weather and COVID-19 was detected in Saudi Arabia, which was similar with previous findings from Bangladesh, the USA, China, Japan and Indonesia (Bashir et al., 2020, Şahin, 2020, Sajadi et al., 2020, Sharif and Dey, 2021, Sharif et al., 2021a, Sharif et al., 2021b, Tosepu et al., 2020) This study included three times of analyses for the parameters of weather following previous studies (Sharif and Dey, 2021, Sharif et al., 2021a, 2021b). In accordance with previous studies, we included about twelve parameters of weather from three time frames (Sharif et al., 2021b). Further, this study analyzed the impact of weather for about two years on COVID-19 and seasonality of the pandemic in Saudi Arabia for the first time. Cases of COVID-19 had the highest correlation with relative humidity (RH) (*r*_s_ = −0.62) and minimum temperature (*r*_s_ = −0.61). The growth rate had the strongest association with sun hours (*r*_s_ = −0.63) and the mortality had the highest correlation with RH on the day (*r*_s_ = −0.55). These findings were in good agreement with previous studies (Bashir et al., 2020, Bilal et al., 2021, Sajadi et al., 2020, Sharif and Dey, 2021, Sharif et al., 2021a, Sharif et al., 2021b, Şahin, 2020, Tosepu et al., 2020). Weather may have intervened with the COVID-19 pandemic by affecting the transmission in different conditions. Extreme temperature, UV and humidity have direct impact on the destruction of virus particles both in environment and laboratory set up (Bashir et al., 2021, Bilal et al., 2021, Sharif and Dey, 2021, Sharif et al., 2021a, Sharif et al., 2021b, Tan et al., 2005, White et al., 2020, Xie and Zhu, 2020). Therefore, increased temperature and UV index can significantly affect transmission of SARS-CoV-2 (Bashir et al., 2020, Şahin, 2020, Sajadi et al., 2020, Tosepu et al., 2020). Previous studies have also demonstrated that temperature >40 °C can significantly reduce the concentrations of SARS-CoV, MERS-CoV in environment (Rabenau et al., 2005, Tan et al., 2005, Van Doremalen et al., 2013). Wind velocity is another important factors that can regulate the concentration of virus particles in both closed-indoor and outdoor environments. In this study we detected positive correlation of wind velocity with the cases and fatalities, which is similar with previous studies in Bangladesh, China, and Japan (Bashir et al., 2020, Bilal et al., 2021, Şahin, 2020, Sajadi et al., 2020, Sharif and Dey, 2021, Sharif et al., 2021a, Sharif et al., 2021b, Tan et al., 2005, Tosepu et al., 2020, White et al., 2020, Xie and Zhu, 2020).

Population factors including population density and total population of a community are great contributors to contain the virus for long time (Sharif and Dey, 2021). Strong positive correlation between case and population density (*r*_s_ = 0.57), case and total population (*r*_s_ = 0.61), fatality and population density (*r*_s_ = 0.71) were detected. These findings are in good agreement with previous studies in Japan and Bangladesh (Sharif and Dey, 2021, Sharif et al., 2021b). In this study, the impact of vaccination of cases and fatalities were also determined. Significant negative correlation was detected between the frequency of vaccination and cases (*r*_s_ = −0.81), vaccination and fatalities (*r*_s_ = −0.85) in Saudi Arabia. These findings indicated that community transmission of COVID-19 are reducing in vaccinated cities.

One peak of cases and fatalities was confined within the period of May 2020 to September and another peak is on the rise from April 2021 in Saudi Arabia. During these seasons the temperature were relatively higher than other seasons. Environmental parameters, human activities, social gatherings, religious and sport events, and local and global migrations and spread of variants with altered transmission rate have influenced the growth of COVID-19 cases and fatalities in Saudi Arabia. However, the seasonal pattern of COVID-19 infection in Saudi Arabia is well determined in this study.

The genomic surveillance of this this study detected that clade GH (54%) was the most prevalent in Saudi Arabia. The phylogenomic analyses revealed that among the variant of concern (VOC), only alpha and beta variants are circulating in Saudi Arabia. Further, this study found that most of the isolates in Saudi Arabia were closely related with B.1.427/429 variant. This is one of the first reports of overall genomic diversity of SARS-CoV-2 in Saudi Arabia.

Numerous substitution point mutations, insertion, deletions and recombination in the gnome of the virus have influenced the emergence of new variants with altered properties (Roy et al., 2021). About 1,000 whole genome were analyzed in this study. However, the frequency of substitution mutations at spike proteins were relatively lower than other structural and non-structural proteins. At spike protein, D614G, H69del, and N501Y were prevalent in study isolates. Cluster mutations at spike protein receptor binding domains (RBD) were not detected in this study. Several host factors including gender, age, population density and case number increased the risks of mutation at S, E, M, N and other nonstructural protein sites. However, increased vaccination was associated with lower risk of mutations both at structural and nonstructural proteins. Mutations at nucleocapsid protein and other nonstructural proteins were also lower in Saudi Arabia. Lower frequency of mutations in the isolates may be due to the lack of enough sequence data in Saudi Arabia.

This study detected that male was the prevalent sex group among the patients. In age distribution analysis, the highest frequency of hospitalizations, critical conditions and fatality were detected among the participants aged >60 years in Saudi Arabia. These findings were similar with the previous studies in Bangladesh, Japan, the USA, Italy, China, Indonesia and many other countries (Sharif and Dey, 2021, Sharif et al., 2021a, Sharif et al., 2021b).

This study reported significant correlation of environmental parameters, host factors and population factors on the frequency of substitution point mutations. We detected strong correlation between sun hours and mutations at nucleocapsid proteins, temperature and spike protein, UV and non-structural proteins. These findings are in good agreement with previous studies in Japan and Bangladesh (Bashir et al., 2020, Bilal et al., 2021, Şahin, 2020, Sajadi et al., 2020, Sharif and Dey, 2021, Sharif et al., 2021a, Sharif et al., 2021b, Tan et al., 2005, Tosepu et al., 2020, White et al., 2020, Xie and Zhu, 2020).

At present, emergence of new variants like VOC delta variant and their rapid transmission with high mortality rate are shaping the wave of the pandemic globally (Sharif et al., 2022). Vaccination and preventive health measures are the best options to minimize the transmission and fatalities associated with the pandemic. In addition to the seasonality, several factors like people tendency to follow preventive measures, previous health conditions and comorbidities in patients, health policy by authorities, international and national movements, vaccination, social and religious events and easy access to test and health facilities have regulated the wave of COVID-19 (Alrashed et al., 2020, Sharif et al., 2021c, Sharif et al., 2021d).

As far as we know, this is the first study to analyze seasonality and impact of meteorological parameters on the COVID-19 pandemic in Saudi Arabia. This study has detected significant impact of environmental factors, population factors and vaccination on COVID-19. We also detected significant impact of host factors and weather on the mutation frequency of SARA-CoV-2. This study created an integrated overview of the circulating variants in Saudi Arabia. However, we detected a significant lack of whole genome data, which should be increased to understand the diversity of variants in Saudi Arabia. This study will provide significant information to evaluate the seasonal increase of COVID-19 in temperate regions for future studies focusing on COVID-19 pandemic. In future, detail analysis of whole genome with their impact on the pandemic should be performed based on the findings in this study to provide a complete scenario of the regulatory factors and their effects on the COVID-19 pandemic.

## 5 Conclusion

This is one of the first studies to analyze and evaluate the correlation of environmental parameters, population factors, and human activities with the COVID-19 pandemic in Saudi Arabia. This study has provided an overall insight into the seasonality of the COVID-19 pandemic during nineteen months in Saudi Arabia. In this study, different regulatory factors of COVID-19 pandemic have been analyzed. We found significant impact of host factors, human activities and environmental parameters on the COVID-19 pandemic in Saudi Arabia. This study reported the circulation of alpha and beta variants in Saudi Arabia in recent times. We also detected lack of whole genome sequencing in Saudi Arabia that could possibly overlook the exact diversity of variants. This study suggests that weather may play a key role in determining the transmission and severity of the pandemic in temperate climate. This study will provide a complete baseline database to the national and international organizations and legislators to explore the COVID-19 pandemic in details in Saudi Arabia.

## Declaration of Competing Interest

The authors declare that they have no known competing financial interests or personal relationships that could have appeared to influence the work reported in this paper.

## Figures and Tables

**Supplementary Fig. 1A–C:**
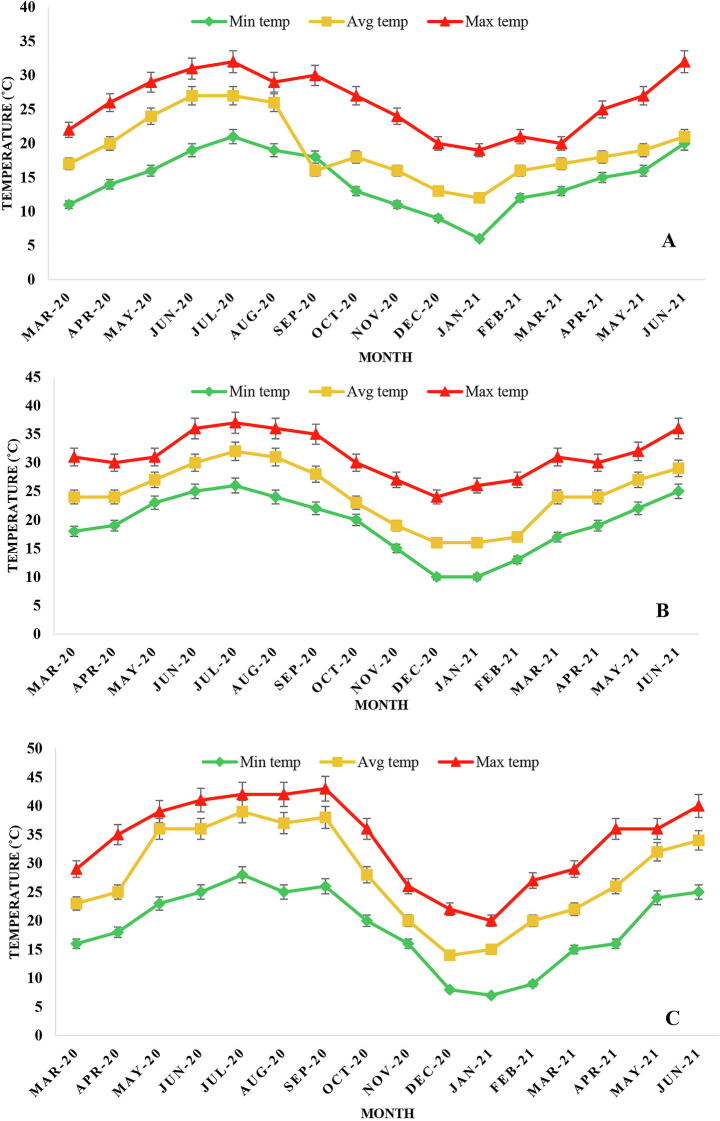
Monthly trends of mean value of temperature minimum (min temp), temperature average (avg temp) and temperature maximum (max temp) in A. Asir; B. Al Bahah; C. Al Jawf during March, 2020 to July, 2021.

**Supplementary Fig. 1D–F:**
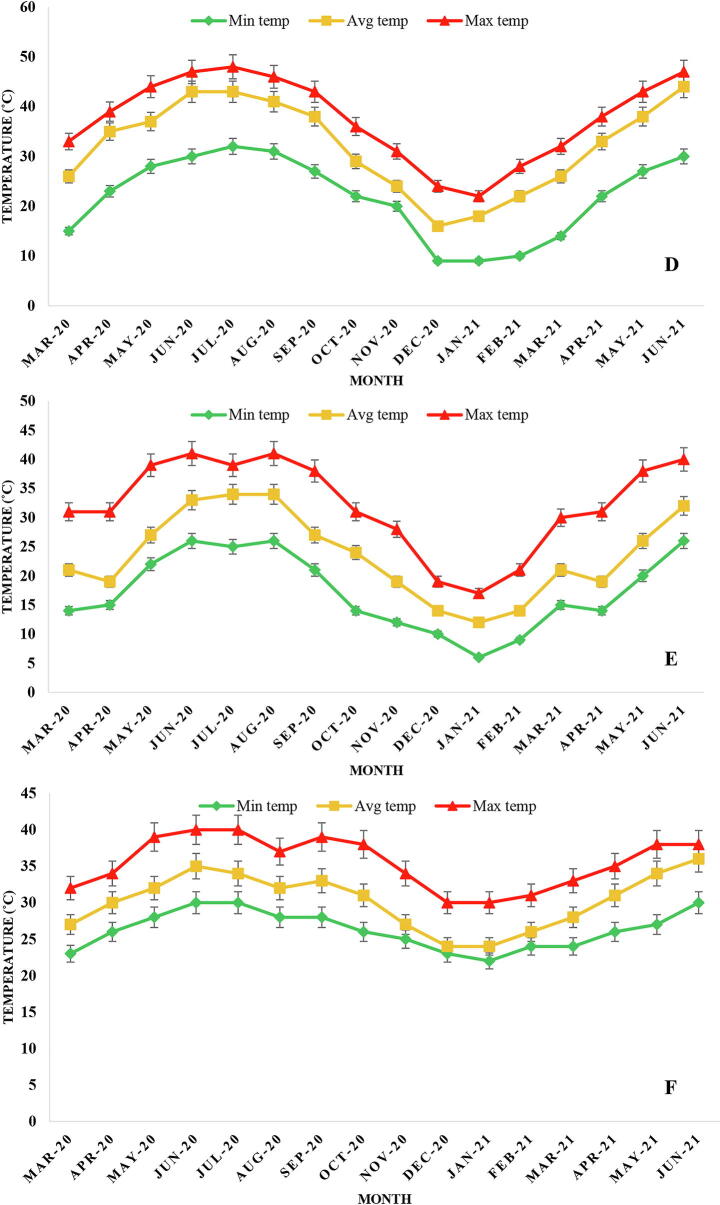
Monthly trends of mean value of temperature minimum (min temp), temperature average (avg temp) and temperature maximum (max temp) in D. Eastern Province; E. Ha'il; F. Jizan during March, 2020 to July, 2021.

**Supplementary Fig. 1G–I:**
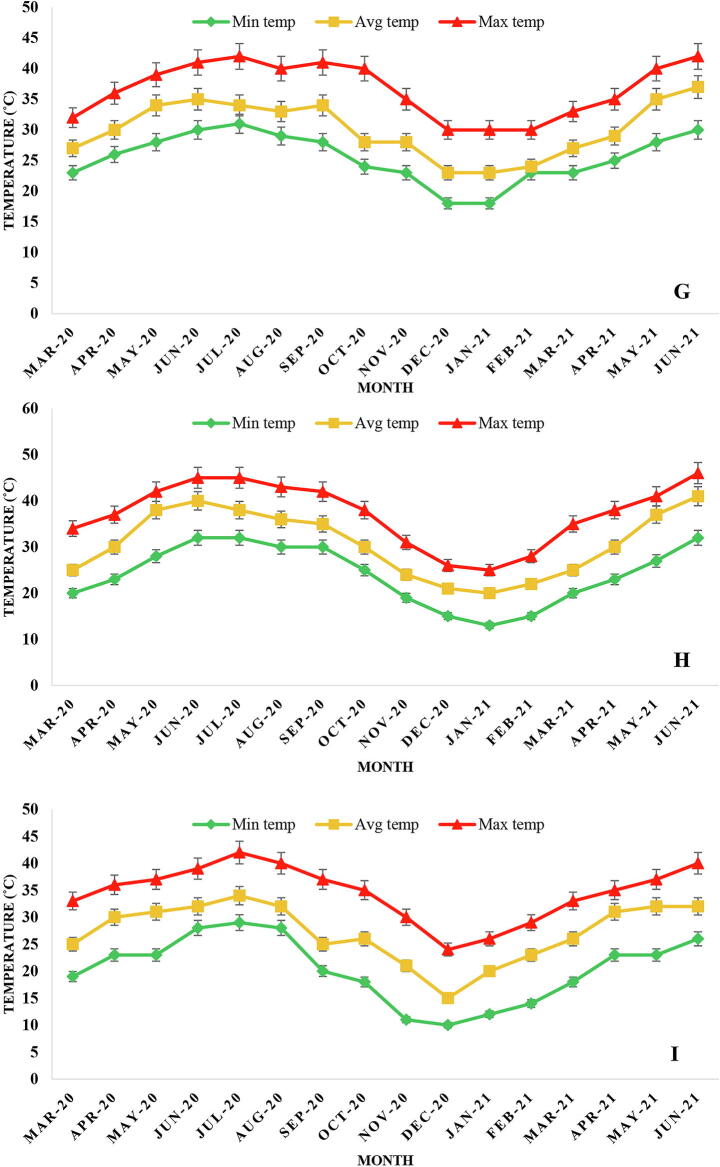
Monthly trends of mean value of temperature minimum (min temp), temperature average (avg temp) and temperature maximum (max temp) in G. Makkah; H. Madinah; I. Najran during March, 2020 to July, 2021.

**Supplementary Fig. 1J–L:**
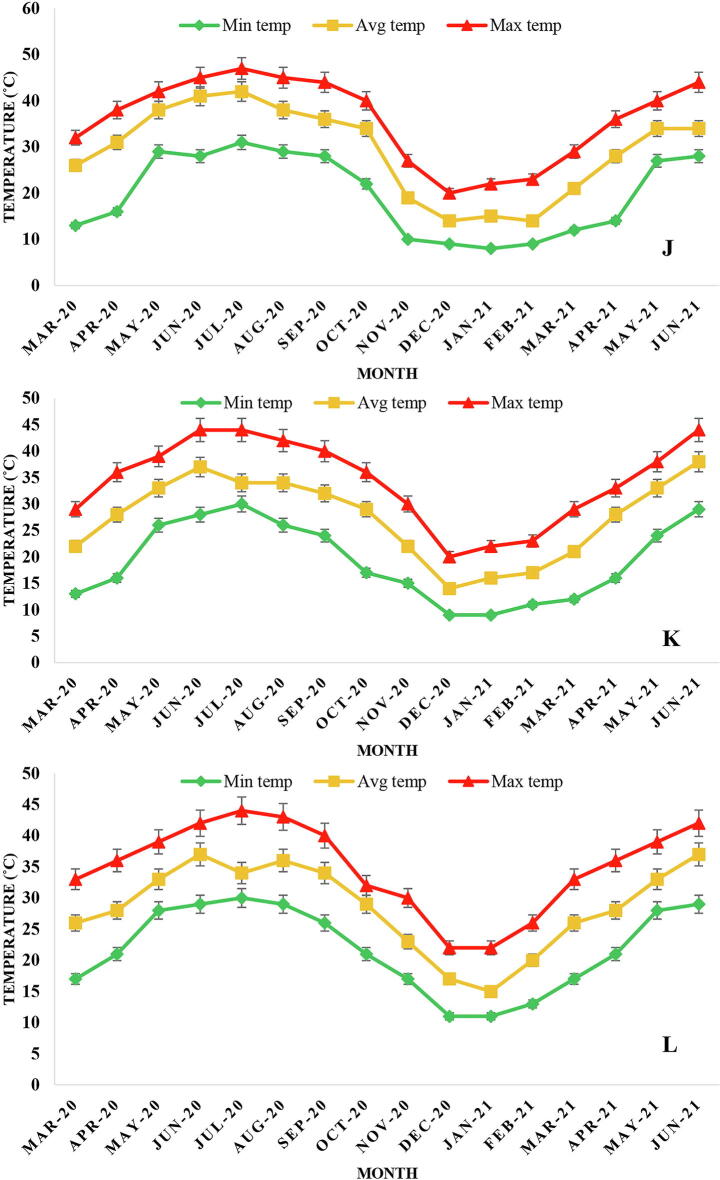
Monthly trends of mean value of temperature minimum (min temp), temperature average (avg temp) and temperature maximum (max temp) in J. Northern Borders; K. Qasim; L. Riyadh; M. Tabuk during March, 2020 to July, 2021.

**Supplementary Fig. 1M:**
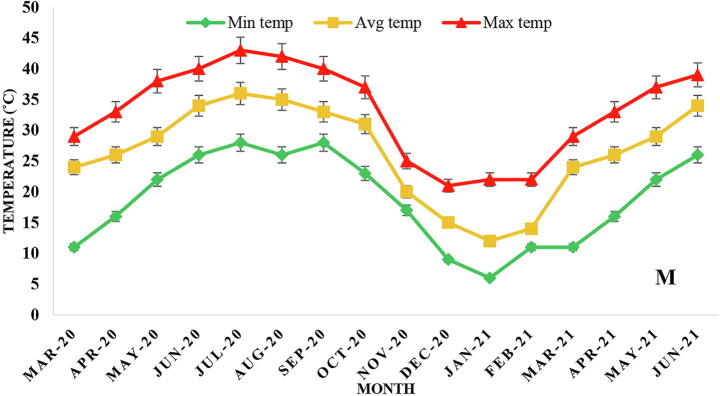
Monthly trends of mean value of temperature minimum (min temp), temperature average (avg temp) and temperature maximum (max temp) in M. Tabuk during March, 2020 to July, 2021.

**Supplementary Fig. 2A:**
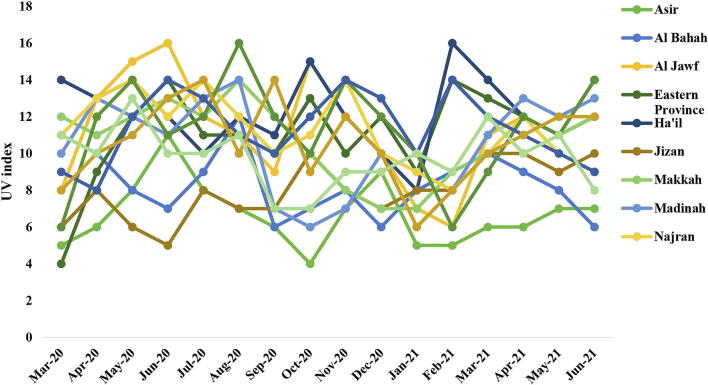
Month-wise trends of average UV index in Saudi Arabia.

**Supplementary Fig. 2B:**
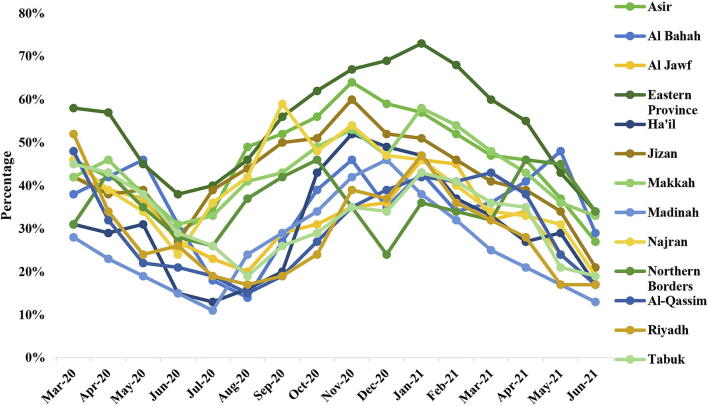
Distribution of average relative humidity (RH) in Saudi Arabia during March, 2020 to July, 2021.

**Supplementary Fig. 2C:**
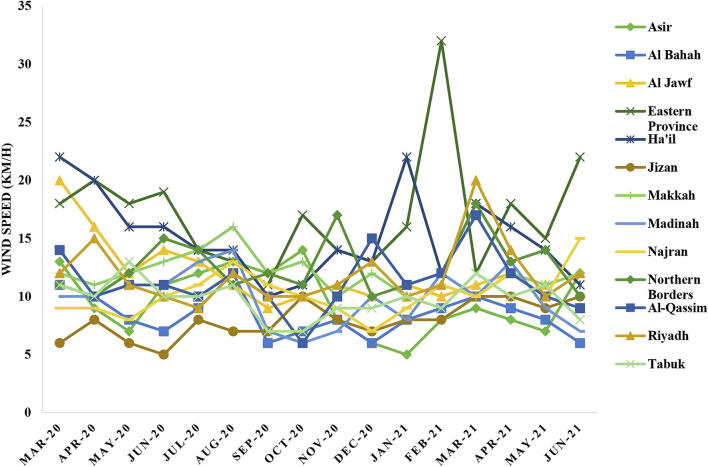
Trends of average wind speed in 13 provinces in Saudi Arabia.
